# Design of Self-Expanding Auxetic Stents Using Topology Optimization

**DOI:** 10.3389/fbioe.2020.00736

**Published:** 2020-07-14

**Authors:** Huipeng Xue, Zhen Luo, Terry Brown, Susann Beier

**Affiliations:** ^1^School of Mechanical and Mechatronic Engineering, University of Technology Sydney, Ultimo, NSW, Australia; ^2^School of Mechanical and Manufacturing Engineering, University of New South Wales, Sydney, NSW, Australia

**Keywords:** self-expanding stents, auxetic metamaterials, topology optimization, level set method, additive manufacturing

## Abstract

Implanting stents is the most efficient and minimally invasive technique for treating coronary artery diseases, but the risks of stent thrombosis (ST) and in-stent restenosis (IRS) hamper the healing process. There have been a variety of stents in market but dominated by *ad hoc* design motifs. A systematic design method that can enhance deliverability, safety and efficacy is still in demand. Most existing designs are focused on patient and biological factors, while the mechanical failures related to stenting architectures, e.g., inadequate stent expansion, stent fracture, stent malapposition and foreshortening, are often underestimated. With regard to these issues, the self-expanding (SE) stents may perform better than balloon-expandable (BE) stents, but the SE stents are not popular in clinic practice due to poor deliverability, placement accuracy, and precise match of the stent size and shape to the vessel. This paper addresses the importance between stent structures and clinic outcomes in the treatment of coronary artery disease. First, a concurrent topological optimization method will be developed to systematically find the best material distribution within the design domain. An extended parametric level set method with shell elements is proposed in the topology optimization to ensure the accuracy and efficiency of computations. Second, the auxetic metamaterial with negative Poisson’s ratio is introduced into the self-expanding stents. Auxetics can enhance mechanical properties of structures, e.g., fracture toughness, indentation and shear resistance and vibration energy absorption, which will help resolve the drawbacks due to the mechanical failures. Final, the optimized SE stent is numerically validated with the commercial software ANSYS and then prototyped using additive manufacturing techniques. Topological optimization gives a rare opportunity to exploiting the unique advantages of additive manufacturing. Hence, the topologically optimized auxetic architectures will provide a new solution for developing novel stenting structures, especially conductive to self-expanding SE stents. The new design will overcome the limitations of conventional SE stents associated with mechanical structures while maintain their valuable features, to help reduce the occurrence of ST and ISR and benefit the clinic practice in treating coronary heart disease.

## Introduction

The concept of angioplasty in 1964 (Dotter and Judkins, 1964) had helped the development of the first balloon coronary angioplasty in 1977 (Gruntzig, 1978). Developments in percutaneous coronary intervention (PCI) technology led to the generation of new treatment methods for coronary artery disease (CAD), namely interventional cardiology (Garg and Serruys, 2010). In order to decrease the rates of restenosis and abrupt closure of arteries (Gruntzig et al., 1979; Venkitachalam et al., 2008), coronary stents were first introduced in PCI after angioplasty (Sigwart et al., 1987). During the early periods, successful results in treating abrupt and susceptible vessel closure showed the effectiveness of coronary stents (Roubin et al., 1992). However, the high rate of subacute thrombotic coronary artery occlusion after implantation significantly hampers their further development and application (Serruys et al., 1991). They were not widely accepted in clinics until the safety of the coronary stents was evidenced by two comparison studies (Fischman et al., 1994; Serruys et al., 1994) between stent implantation and balloon angioplasty. After that, most PCI procedures adopted coronary stent implantation. The obvious advantage of stent implantation is the providing of an effective and continuous support for clogged arteries with minimal invasion. Despite the above advances, the risks of stent thrombosis (ST), in-stent restenosis (ISR), and other complications still exist and impact their safety evolution (O’Brien and Carroll, 2009).

It is hard to prevent ST and ISR at the same time for stents at today’s market. The implantation of stents will cause accumulation of macrophages around stents, which lead to neointimal proliferation of nearby smooth muscle cells and the new tissue growth still occurs in response to the injury, finally result in ISR. This proliferation can be greatly limited by drugs released from drug-eluting stents (DES) (Stettler et al., 2007; Stone et al., 2007), but the drugs also prevent the formation of a new endothelial layer which can effectively inhibit clot formation when compared to the bare-mental stents (BMS). In the vascular healing, endothelialization plays an important role to prevent the formation of thrombus. Hence, the first-generation of DES has been evidenced to be able to effectively reduce the ISR incidence by 5–15% at 12 months, compared to 20–30% incidence in BMS (Torrado et al., 2018), but it is reported to trigger an increased incidence of ST (up to 2%) (Palmerini et al., 2015) at the later stage after implantation, while the much cheaper BMS suffer an opposite effect (1.2% in ST) (Torrado et al., 2018). These issues result in the development of seeking new polymer coatings, antiproliferative drugs, and materials of stent platform, such as biodegradable or bioresorbable materials (McMahon et al., 2018; Siiki et al., 2018). However, several ABSORB trials of biodegradable stents (BDS) (Kang et al., 2014) and bioresorbable vascular scaffolds (BVS) demonstrate inferior outcomes, evidenced by the fact that the first type of commercial biodegradable stents were withdrew from the market in 2017. These developments are successfully in control ISR in short-term, especially for the second-generation DES with less than 5% incidence within 12 months, the high-profile BDS and BVS are recently still limited by their materials and present a slight higher ST incidence, 0.5–1.3% and 1.4%, respectively, compared with 0.7% in the second-generation DES (Kalra et al., 2017). Although the second generation DES are superior to other current stents and recommended in the clinic use, the long heal time are still facing late stent failure that may result in an increased occurrence of ISR and ST (Torrado et al., 2018). ST is a complex multifactor pathophysiology, and it is a rare complication but with high mortality (5–45%), relapse rate (15–20%) at 5 years (Gori et al., 2019). ISR is also a concerned issue when facing long-term treatments. Hence, the risk of stent thrombosis and restenosis still demands new development of stents.

Apart from patient and biological reasons, the mechanical or procedural factors also play in important role in the incidence of ST and ISR, e.g., inadequate stent expansion, stent fracture, late stent malapposition and foreshortening. These issues due to stenting structural and procedural issues occur no matter what kind of stents are employed. Neointimal hyperplasia will occur once the gap exists between the struts and the vessel wall, which may lead to a risk of ISR (Foin et al., 2014). There has been evidence that these mechanical factors were associated with late or very late ST (Gori et al., 2019). Based on the mechanism of stenting expansion, stent struts can be divided into self-expanding (SE) and balloon-expandable (BE). With respect to the above issues relevant to mechanical structures, SE stents show certain superiority than BE stents.

SE stents are often constrained within a delivery catheter until positioned and deployed. It expands spontaneously when released from the constraining device, and several different mechanisms can be used to achieve “self-expanding,” such as shape memory property of materials and mechanical “spring-like” design to achieve expansion. After implantation with a full deployment, they position themselves against the vessel wall with a slowly released but continuously supported outward force. They are normally characterized with a less hoop strength that can be defined as the resistive force to radial compressive forces, in comparison with BE stents. SE stents are usually made of superelastic materials, such as Nitinol, an alloy of nickel and titanium with excellent corrosion resistance and biocompatibility, and nitinol SE stents are manufactured to have a size slightly larger than the target vessel size.

Although SE stents are not as popular as BE stents in application, it has a strong ability in some atypical and complex coronary anatomy (Lu et al., 2018; Pellegrini and Cortese, 2019), demonstrated by the successful clinic use of commercial product Stentys^®^ Self-Apposing^®^ stent. The benefits of SE stents can be summarized as follow: (1) the gradual expansion process of SE stents results in no-reflow and a lower incidence of edge dissections, as well as avoidance of inadequate stent expansion (Kidawa et al., 2017); (2) the avoidance of immediate vessel wall injury, leading to a lower injury response of the artery, so as to reduce the incidence of acute thrombosis, as well as neointimal hyperplasia for ISR and a larger lumen area (Schmidt and Abbott, 2018); (3) continuously supplying radial outward force to support vessel wall to prevent the occurrence of late stent malapposition and the complications (Kidawa et al., 2017; Lu et al., 2018). Hence, SE stents can effectively support the enlarged vessel after the disruption of plaques; and (4) the supply of better flexible deformation, especially for bifurcations and vessels with a significant tapering (Pellegrini and Cortese, 2019) due to material elasticity rather than the permanent deformation of plasticity from BE stents, leading to a low fracture incidence. More introduction for self-expanding stents may refer to https://www.sciencedirect.com/topics/nursing-and-health-professions/self-expanding-stent.

However, the current development and application of SE stents are significantly hindered by some drawbacks. The first is the relatively large profile of the catheter that makes the delivery system cumbersome, which increases the difficulty of delivering and the risk of injury during percutaneous puncture. The second is a complicated procedure caused by the phenomenon of stent foreshortening that lows the deployment accuracy (Schmidt and Abbott, 2018). When expands, the superelasticity of SE stents often comes with the cost of a foreshortening such as 20% of the undeployed length, which makes positioning difficult. The deviation of deployment can lead to uncovered lesions, resulting in a high risk of thrombosis complications. The third is the precise requirement of matching the stent shape to the target vessel shape. In practice, the diameter of a SE stent can hardly be precisely evaluated due to the complexity of real-world clinic cases, as an over-small size may cause stent malapposition, and an over-large size may lead to a larger lumen even a negative late loss of stent (Schmidt and Abbott, 2018). Stent malapposition, or incomplete stent apposition, is a morphological description used to indicate one or more stent struts that do not contact the intimal surface of vessel wall tightly. Stent malapposition is of great importance, as it may increase the risk of subsequent stent thrombosis.

With respect to most up-to-date stents (both SE and BE stents), the importance between stenting structural designs and their clinic outcomes in the treatment of coronary artery disease has been underestimated. Furthermore, the current designs for seeking new generations of stents are more focused on the improvement of stent performance based on material properties and biological aspects. However, a stent performance depends on not only the new materials and biological factors but also stenting structural architectures. The stents are first-of-all both a mechanical and biological structure to expand clogged arteries with appropriate elasticity and flexibility, while simultaneously withstand the radial compressive forces with a prescribed hoop strength.

Since finite element method (FEA) provides an efficient computational tool to evaluate mechanical properties of stents (Karanasiou et al., 2017; Chen et al., 2020), more and more studies began to focus on the design of stent with FEA (Bressloff et al., 2016; Blair et al., 2019). For instance, surrogate modeling (Putra et al., 2019) is widely used to perform size or shape optimizations for stent structs. The objective function include dogboning (Chen et al., 2019), foreshortening (Torki et al., 2020), flexibility (Shen et al., 2019), radial stiffness (Torki et al., 2020), recoil (Li et al., 2016), fatigue strength (Alaimo et al., 2017), and haemodynamic disturbance (Prithipaul et al., 2018; Putra et al., 2019). The design parameters are usually the thickness, link width and length, strut width and length, curve radius, profile shape, and pattern numbers. However, no matter what kind of surrogate models are used, size optimization gives a narrow space to improve stent performance, as the design only depends on size parameters under a given shape and topology.

In this paper, we will develop a more effective systematic design method to find new stenting structures for future generation of SE stents, with a view to improving stent performance by optimizing stenting structure of topology and shape. The new designs for stenting structural architectures will eventually improve stent safety to low the incidence of restenosis and thrombosis. Hence, to help overcome the above drawbacks in current SE stents, this paper will firstly introduce the auxetic microstructures (Lakes, 1993; Evans and Alderson, 2000) into SE stents to achieve closed-cell stents, and then develop an enhanced topological optimization method with level sets (Luo et al., 2007; Li et al., 2016), which is more accurate and more efficient in finding the best architecture for auxetic microstructures.

Auxetics are a family of mechanical metamaterials, with microstructures artificially engineered to have unusual elasticity property, namely, negative Poisson’s ratio (NPR). Auxetics contract in transverse direction when compressed in axial direction and vice versa. Auxetics provide potential for a wide range of applications in different fields, due to their unique properties for energy absorption, anti-impact, indentation resistance, thermal isolation, and fracture toughness. Auxetics will particularly benefit the SE stents from the following aspects:

(1) Auxetic behavior can help make the size of SE stents much smaller than current profile of SE stents. Small profiles of SE stent systems will facilitate deliverability, and therefore reduce the occurrence of complications due to potential injury in the process of percutaneous coronary intervention. After implantation, they fully stretched themselves and adaptively fit the vessel wall. This will help reduce the potential injury to arterial intimal surface, leading to a low injury response and therefore restenosis.

(2) The effective NPR property of auxetic microstructures makes SE stents have non-shortening that facilitates accurate sent deployment when deployed, which further reduces stent malapposition. As we know, stent implantation often leads to suboptimal results, e.g., the occurrence of strut malapposition, especially in cases of complex lesions and other factors. The indentation resistance of auxetics enables a superior conformability of stent struts to automatically match the vessel wall surface, allowing the stent to contact the vessel surface perfectly without foreshortening and malapposition.

(3) The auxetic microstructures can adaptively respond to different radial compressive force, thus make the auxetic stent have variable hoop strengths that allows the stent to easily adapt complex shapes in interventional cardiology such as tortuous and clogged arteries. Coronary stent size matters. In response to large artery size and small radial force, the auxetic stent will automatically change its shape, and it will continuously expand outwardly and reduce its radial resistance force, to avoid malapposition. Otherwise, when the auxetic stent subject to a small artery size and large hooping force, the stent will increase its structural stiffness and strength (due to indentation resistance) to withstand an increased radial compressive force applied to the stent from the vessel. The adaptive stiffness and strength provided by the auxetic stents will help reduce the risk of restenosis and thrombosis.

(4) The auxetics can also improve stenting mechanical performance and reduce the happening of stent fracture and enhance their ability to withstand fatigue and vibration, because the NPR property can greatly improve the fracture toughness of structures. This will also benefit a low incidence of restenosis.

Topology optimization has been a popular option to realize the optimization of shape and topology of geometries, because it is a numerical procedure that can iteratively find the best material distribution in the design domain. Few studies have attempted to introduce topology optimization into the design of stents, such as (James and Waisman, 2016), but the topology optimization was only used for a bi-stable design during the expansion of the stent to eliminate the axial displacement. There has been no previous work that systematically integrates topology optimization with auxetics to develop auxetic SE stents, and no work has employed multiscale concurrent topology optimization method to create novel stenting structures. Hence, topology optimization can find the optimized performance of stenting structures, associated with the best material layout and the most efficient material usage in the design. Particularly, the concurrent multiscale topology optimization method, with X-PLSM and the numerical homogenization method, can systematically integrate the NPR mechanical metamaterials into the SE stent to implement a new mechanism for expanding, by fully making use of the auxetic behavior of microstructures. The topologically designed, micro-structured, multiscale cellular composite structure is characterized with small size, large expansion, uniform radial force and super compliance, helping avoid the injury to vessel surface, edge-dissection and side-branch, to low thrombosis and restenosis.

## Design Method

In this section, the X-PLSM and the numerical homogenization method is combined as a system to conduct the heuristic multiscale concurrent topology optimization for design of auxetic SE stents.

### Numerical Homogenization Method

The homogenization (Bendsøe and Kikuchi, 1988; Suzuki and Kikuchi, 1991) technique assumes that the design domain is composed of periodic configuration of unit cells, which are much smaller that the bulk material in size. Based on that concept, in this paper, the numerical homogenization method will be used to roughly estimate the effective properties of micro/meso structures. For instance, the effective elasticity tensor *DH ijkl* can be calculated by:

(1) $$D_{i\,j\,k\,l}^{H}=\frac{1}{\left|\Omega \right|}\,\int_{\Omega }\left(\varepsilon_{p\,q}^{0\,\left(i\,j\right)}-\varepsilon_{p\,q}^{*\left(i\,j\right)}\right)\,D_{p\,q\,r\,s}\,\left(\varepsilon_{r\,s}^{0\,\left(k\,l\right)}-\varepsilon_{r\,s}^{*\left(k\,l\right)}\right)\,d\Omega$$

where *i*, *j*, *k*, *l* and *p*, *q*, *r*, *s* are all used to denote 1, 2. *Ω* is the design domain; | *Ω*| is the area of the design domain; *D*_*pqrs*_ is the elasticity tensor of the base material; *ε0(ij) pq* is the test unit strain field, where (1,0,0)*^*T*^*, (0,1,0)*^*T*^* and (0,0,1)*^*T*^* are used in two-dimensional cases; *ε^∗^(ij) pq* is the locally varying strain field due to the application of the unit strain field, which is defined by:

(2) $$\varepsilon_{p\,q}^{*\left(i\,j\right)}=\varepsilon_{p\,q}^{*}\,\left(u^{\left(i\,j\right)}\right)=\frac{1}{2}\,\left(u_{p,q}^{\left(i\,j\right)}+u_{q,p}^{\left(i\,j\right)}\right)$$

By using the finite element method with periodic boundary conditions, the displacement field *u*^(ij)^ can be calculated by:

(3) $$\int_{\Omega }\left(\varepsilon_{p\,q}^{0\,\left(i\,j\right)}-\varepsilon_{p\,q}^{*}\,\left(u^{\left(i\,j\right)}\right)\right)\,D_{p\,q\,r\,s}\,\varepsilon_{r\,s}^{*}\,\left(v^{\left(k\,l\right)}\right)\,d\Omega =0,\forall v^{\left(k\,l\right)}\in \bar{U}\,\left(\Omega \right)$$

where *ν^(kl)^* is the virtual displacement field in*−U*(*Ω*), which denotes the space of all the kinematically admissible displacements in the design domain *Ω*.

### Level Set-Based Parameterization Method

**FIGURE 1:**
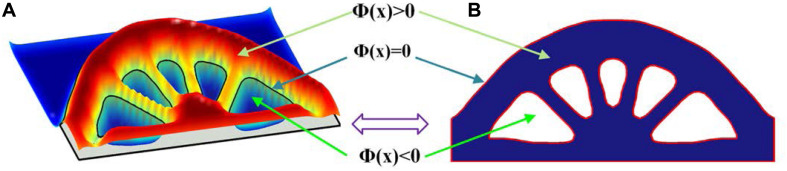
**(A)** 3D level set surface; **(B)** 2D level set boundary.

Topology optimization is used to perform the optimization of the periodic microstructures. The most popular topological optimization methods includes the SIMP method (Solid Isotropic Material with Penalization) (Zhou and Rozvany, 1991; Bendsøe and Sigmund, 1999), and the Level set method (Sethian and Wiegmann, 2000; Wang et al., 2003; Allaire et al., 2004). The level set method adopts an implicit description scheme, to embed the design boundaries of the structure into the zero level set of a higher-dimensional level set function, where an illustration of 2D example is presented in Figure 1.

(4) $$\left\{ \begin{array}{c} \Phi \,\left(x\right)>0\;x\in \Omega \backslash \partial \Omega \;\left(M\,a\,t\,e\,r\,i\,a\,l\right)\\ \Phi \,\left(x\right)=0\;x\in \partial \Omega \;\left(B\,o\,u\,n\,d\,a\,r\,y\right)\\ \Phi \,\left(x\right)<0\;x\in D\backslash \left(\Omega \,⋃\partial \Omega \right)\;\left(V\,o\,i\,d\right) \end{array}\right.$$

where *x* is the point in the space *D*. *Ω* and *∂Ω* denote the design domain and the boundary, respectively.

Since the optimization is driven by the evolution of the level set function, shape and topology of the structure with clear boundaries can be achieved at the same time. However, most conventional level set methods require complicated numerical implementations, e.g., the re-initialization, extension of the boundary velocity field and CFL condition. To overcome these limitations, several alternative methods have been developed, such as the parametric level set method (PLSM) (Luo et al., 2007, 2009) based on compactly supported radial basis functions (CSRBFs) has been successfully applied in dealing with designs in mechanical metamaterials (Wang et al., 2014; Wu et al., 2017; Li et al., 2018).

In the conventional level set method, the optimization process is described as the dynamic motion of the level set function *Φ*(*x*). By introducing a pseudo time *t*, this dynamic change can be determined using Eq. (5), which is actually the Hamilton–Jacobi partial differential equation (PDE). Thus, the optimization is transferred into a procedure to find an appropriate velocity field *v*_*n*_ to solve the Hamilton–Jacobi PDE.

(5) $$\frac{\partial \Phi \,\left(x,t\right)}{\partial t}-v_{n}\,\left|\nabla \Phi \,\left(x,t\right)\right|=0$$

Within the PLSM, the level set function is calculated through centrally positioning CSRBFs at a set of given knots over the whole design domain. That interpolation method is as follows:

(6) $$\Phi \,\left(x,t\right)=\varphi \,{\left(x\right)}^{T}\,\alpha \,\left(t\right)=\sum_{i=1}^{N}\varphi \,\left(x\right)\,\alpha_{i}\,\left(t\right)$$

where *N* is the number of fixed knots in the design domain. The vector with the CSRBFs functions is:

(7) $$\varphi \,\left(x\right)={\left[\phi_{1}\,\left(x\right),\phi_{2}\,\left(x\right),\ldots ,\phi_{N}\,\left(x\right)\right]}^{T}$$

and the expansion coefficient vector is given by:

(8) $$\alpha \,\left(t\right)={\left[\alpha_{1}\,\left(t\right),\alpha_{2}\,\left(t\right),\ldots ,\alpha_{N}\,\left(t\right)\right]}^{T}$$

the CSRBFs of the ith knot used with C2 continuity is given as:

(9) $$\phi_{i}\,\left(x\right)=m\,a\,x\,\left\{0,{\left(1-r_{i}\,\left(x\right)\right)}^{4}\right\}\,\left(4\,r_{i}\,\left(x\right)+1\right)$$

and *r*_*i*_(*x*) is defined as:

(10) $$r_{i}\,\left(x\right)=d_{I}\,/\,d_{m\,I}=\sqrt{{\left(x-x_{i}\right)}^{2}+{\left(y-y_{i}\right)}^{2}}\,/\,d_{m\,I}$$

where *d*_*I*_ denotes the distance between the current sample knot (*x*, *y*) and the ith knot (*x*_*i*_, *y*_*i*_), and *d*_*mI*_ is the influence domain of the knot (*x*, *y*), which means only the knots in that domain can affect the current CSRBFs function.

Since all the RBF knots are fixed in the design domain, this interpolation separates the time and space from the level set function. The original level set function *Φ*(*x,t*) is now determined by the spatial functions *φ*(*x*) located at the knots and the temporal only expansion coefficient α(*t*). The PDE-based level set model is transformed into the following ODE (ordinary differential equation) system:

(11) $$\varphi \,{\left(X\right)}^{T}\,\dot{\alpha }\,\left(t\right)-v_{n}\,\left|{\left(\nabla \varphi \right)}^{T}\,\alpha \,\left(t\right)\right|=0$$

Hence, the normal velocity field are given as:

(12) $$v_{n}=\frac{\varphi \,{\left(X\right)}^{T}}{\left|{\left(\nabla \varphi \right)}^{T}\,\alpha \,\left(t\right)\right|}\,\dot{\alpha }\,\left(t\right),\mathrm{where}\,\dot{\alpha }\,\left(t\right)=\frac{d\,\alpha \,\left(t\right)}{d\,t}$$

### Extended Parametric Level Set Method

Most stenting structures can be regarded as thin-walled structures. The thickness of most stents is around 100 μm, much smaller than sizes of width and length that are usually 4 and 10 mm, respectively. Therefore, the shell element is more suitable for approximating structures of stents. A shear deformable shell element with four nodes is used in this paper. Each element has four nodes and each node has three degrees of freedom *w*_*i*_, *θ_*xi*_* and *θ_*yi*_*, as shown in (13). This kind of element is more convenient and accurate to capture deformation of thin-walled stenting structure. When preforming finite element analysis, the element stiffness matrix is assembled by two parts as illustrated in (14): bending loads calculated by elasticity tensor *D*_*b*_ and shear deformation calculated by *D*_*s*_.

(13) $$q_{i}={\left[w_{i}\,\theta_{x\,i}\,\theta_{y\,i}\right]}^{T},\mathrm{where}\,\theta_{x\,i}=\frac{\partial w_{i}}{\partial y}\,\mathrm{and}\,\theta_{x\,i}=-\frac{\partial w_{i}}{\partial x}$$

(14) $$\left[D_{b}\right]=\frac{E\,h^{3}}{12\,\left(1-\mu^{2}\right)}\,\left[\begin{array}{ccc} 1 & \mu & 0\\ \mu & 1 & 0\\ 0 & 0 & \left(1-\mu \right)\,/\,2 \end{array}\right],\text{and}\,\left[D_{s}\right]=k\,h\,\left[\begin{array}{cc} G & 0\\ 0 & G \end{array}\right]$$

where, *E* and *G* are Young’s modulus and shear modulus, respectively. *μ* is Poisson’s ratio. *k* is the shear energy correction factor; and *h* is the thickness of the shell element.

The numerical implementation method based on zero iso-contour used in the level set method can be utilized to solve a curved shell model, but it has higher computational cost. The level set function (4), can also be described as Eq. (15), where the local velocity *v* can be calculated by three components in the Cartesian coordinates.

(15) $$\frac{\partial \Phi \,\left(x,t\right)}{\partial t}-v\cdot \nabla \Phi \,\left(x,t\right)=0,\mathrm{where}\,v\equiv \frac{\partial x}{\partial t}=\left[v_{x},v_{y},v_{z}\right]$$

**FIGURE 2:**
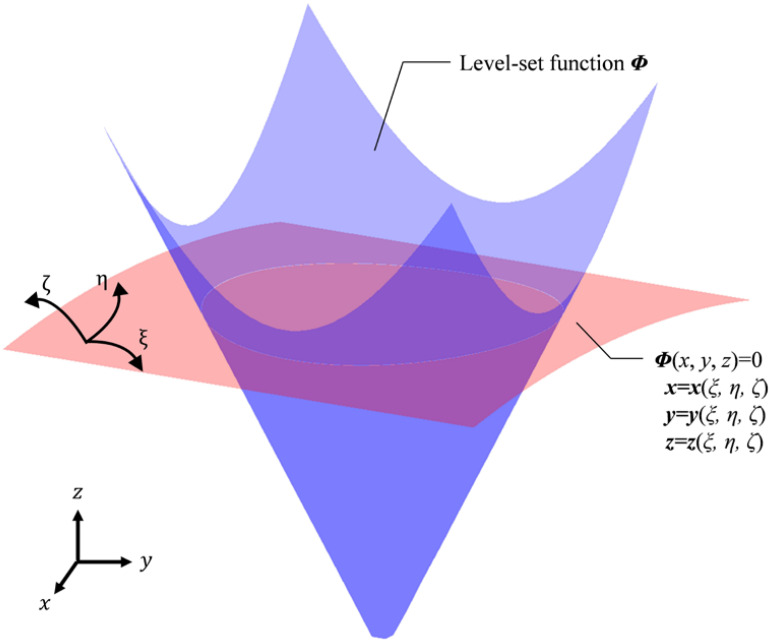
Level set function in curvilinear coordinates.

Hence, if the velocity components are transferred in curvilinear coordinates, the evolution of the level set function can also be described with curvilinear coordinates. As an illustration in Figure 2, the blue color shows a level set function, while the white intersection line is the boundary of a four-node shell structure, located at the zero-level set which is presented as the red curved surface.

Initially, this kind of transformation rule was used to deal with fluid issues (Horiuchi et al., 2004; Yue et al., 2005). Park and Youn (2008) introduced this transformation into level-set topology optimization. Based on the above works, the transformation of the level set function from Cartesian coordinates to curvilinear coordinates can be described as:

(16) $$\frac{\partial \Phi }{\partial t}+\frac{1}{J}\frac{\partial }{\partial \xi^{j}}\left(\frac{J}{\sqrt{g_{j\,j}}}\Phi v_{\xi }^{j}\right)=0\left(j=1,2,3\right),\mathrm{where}\sqrt{g_{j\,j}}=\sqrt{g_{j}\cdot g_{j}}$$

where, *J* = det[*J*], and *J* is the Jacobian matrix; *g*_*j*_ is the covariant basis vector in curvilinear coordinates *ξ ^*j*^*([*ξ*, η, ζ]), and *v j ξ*is the velocity component in the *ξ ^*j*^*-coordinate direction.

Then, we propose an extended PLSM (X-PLSM) by applying this transformation rule into the PLSM based on CSRBFs (Luo et al., 2007). Since the time-derivative is only related to the expansion coefficient α(*t*) in PLSM, the transformation can be applied only for the spatial functions *φ(x)*, as shown in (17), where the subscript *ξ* donates parameters in the curvilinear coordinates *ξ ^*j*^*([*ξ*, η, ζ]), and *φ_ξ_ (ξ)* is the transformed spatial functions.

(17) $$\Phi_{\xi }\,\left(\xi ,t\right)=\varphi_{\xi }\,{\left(\xi \right)}^{T}\,\alpha_{\xi }\,\left(t\right)=\sum_{i=1}^{N}\varphi_{\xi }\,\left(\xi \right)\,\alpha_{\xi ,i}\,\left(t\right)$$

Then, the level set model in Eq. (11) can be rewrite in the curvilinear coordinates, as Eq. (18).

(18) $$\varphi_{\xi }\,{\left(\xi \right)}^{T}\,{\dot{\alpha }}_{\xi }\,\left(t\right)-v_{\xi }^{n}\,\left|{\left(\nabla \varphi_{\xi }\right)}^{T}\,\alpha_{\xi }\,\left(t\right)\right|=0$$

where, *v n ξ*is the normal velocity field in the curvilinear coordinates, and can be given by:

(19) $$v_{\xi }^{n}=\frac{\varphi_{\xi }\,{\left(\xi \right)}^{T}}{\left|{\left(\nabla \varphi_{\xi }\right)}^{T}\,\alpha_{\xi }\,\left(t\right)\right|}\,{\dot{\alpha }}_{\xi }\,\left(t\right),\mathrm{where}\,{\dot{\alpha }}_{\xi }\,\left(t\right)=\frac{d\,\alpha_{\xi }\,\left(t\right)}{d\,t}$$

## Heuristic Concurrent Multiscale Method

In this paper, the objective of the design is to obtain a structure with both auxetic behavior and the stiffness to support vascular walls. Therefore, a concurrent topology optimization strategy is adopted to achieve the design of the auxetic structure in the micro scale and meet the compliance requirement in the macro scale. Since the structure of a stent should be composed of periodic unit cells in a scale that is much bigger than the real microscale, the concept of multiscale in this paper is a kind of heuristic model. In this heuristic multiscale model, the auxetic property is still obtained in a micro scale, but the macrostructure is periodically composed of auxetic microstructures. The realization and relevant sensitivity analysis will be discussed in this section.

### Optimization Model

Initially, the effective elasticity tensor of the microstructure is firstly calculated, and then the Poisson’s ratio is evaluated based on the effective elasticity tensor. Then, the objectives in macro and micro scales are calculated, respectively. After that, they are normalized and assembled by weight factors. Meanwhile, the final sensitivity of multiscale is obtained in the same way. Finally, X-PLSM is used to update the coefficients of the interpolation and so the structural shape and topology. In this optimization, the macrostructures are configured by a series of uniform microstructures. The overall concurrent topology optimization using X-PLSM is formulated as:

(20) $$\left\{ \begin{array}{c} Find\alpha_{\xi ,n}^{M\,I}\left(n=1,2,\ldots ,N\right)\\ M\,i\,n\;J\,\left(\alpha_{\xi }^{M\,I}\right)=W_{1}\,J^{M\,A}\,\left(\alpha_{\xi }^{M\,I}\right)+W_{2}\,J^{M\,I}\,\left(\alpha_{\xi }^{M\,I}\right)\\ =W_{1}\,\left(\frac{1}{2}\,\int_{\Omega_{\xi }^{M\,A}}{\left(u_{\xi }^{M\,A}\right)}^{T}\,K_{\xi }^{M\,A}\,\left(D_{i\,j\,k\,l}^{H}\,\left(D_{i\,j\,k\,l}^{b\,H}\,\left(\alpha_{\xi }^{M\,I}\right),D_{s}\right)\right)\,u_{\xi }^{M\,A}\,d\Omega_{\xi }^{M\,A}\right)\\ +W_{2}\,\left({\left(\mu_{1}\,\left(D_{i\,j\,k\,l}^{b\,H}\,\left(\alpha_{\xi }^{M\,I}\right)\right)+1\right)}^{2}+{\left(\mu_{2}\,\left(D_{i\,j\,k\,l}^{b\,H}\,\left(\alpha_{\xi }^{M\,I}\right)\right)+1\right)}^{2}\right)\\ S.t.\;V\left(\alpha_{\xi }^{M\,I}\right)=\int_{\Omega_{\xi }^{M\,I}}H\left(\Phi_{\xi }^{M\,I}\left(\alpha_{\xi }^{M\,I}\right)\right)d\Omega_{\xi }^{M\,I}-V_{\xi }^{m\,a\,x}\leq 0\\ F_{\xi }^{M\,I}\,\left(u_{\xi }^{M\,I},w_{\xi }^{M\,I},\alpha_{\xi }^{M\,I}\right)=L_{\xi }^{M\,I}\,\left(w_{\xi }^{M\,I},\alpha_{\xi }^{M\,I}\right),\forall w_{\xi }^{M\,I}\in \bar{U}\,\left(\Omega_{\xi }^{M\,I}\right)\\ F_{\xi }^{M\,A}\,\left(u_{\xi }^{M\,A},w_{\xi }^{M\,A},D_{i\,j\,k\,l}^{H}\right)=L_{\xi }^{M\,A}\,\left(w_{\xi }^{M\,A}\right),\forall w_{\xi }^{M\,A}\in \bar{U}\,\left(\Omega_{\xi }^{M\,A}\right)\\ \alpha_{\xi ,m\,i\,n}^{M\,I}\leq \alpha_{\xi ,n}^{M\,I}\leq \alpha_{\xi ,m\,a\,x}^{M\,I} \end{array}\right.$$

where, the superscript “*MA***”** denotes the parameters in the macro scale, and “*MI***”** in the micro scale. The subscript *ξ* denotes the parameters in the curvilinear coordinates *ξ ^*j*^*([*ξ*, η, ζ]). *N* is the total number of fixed knots in the micro design domain. The expansion coefficients of the CSRBF interpolation *αMI ξ, n* are the design variables in the micro scale, ranging between *αMI ξ, min* and *αMI ξ, max*. *J* is the equivalent objective function, comprised of the macro compliance *J*^*MA*^ and micro Poisson’s ratio *J*^*MI*^, where *W*_1_ and *W*_2_ are corresponding weight factors. *V* is the volume constraint and the upper limitation is defined as *Vmax ξ*. *H* is the Heaviside function (Wang et al., 2003) used to denote void and solid materials, given by:

(21) $$H\,\left(\Phi_{\xi }^{M\,I}\right)=\left\{ \begin{array}{cc} \Theta & \Phi_{\xi }^{M\,I}<-\Delta \\ \frac{3\,\left(1-\Theta \right)}{4}\,\left(\frac{\Phi_{\xi }^{M\,I}}{\Delta }-\frac{{\left(\Phi_{\xi }^{M\,I}\right)}^{3}}{\Delta^{3}}\right)+\frac{1+\Theta }{2} & -\Delta \leq \Phi_{\xi }^{M\,I}\leq \Delta \\ 1 & \Phi_{\xi }^{M\,I}>\Delta \end{array}\right.$$

where *Θ* is a small positive number to avoid the singularity of the element stiffness matrix, and Δ is the width for the numerical approximation of *H*. *δ* is the Dirac function which is the derivative of the Heaviside function *H*, and it can be described as:

(22) $$\delta \,\left(\Phi_{\xi }^{M\,I}\right)=\left\{ \begin{array}{cc} \frac{3\,\left(1-\Theta \right)}{4\,\Delta }\,\left(1-\frac{{\left(\Phi_{\xi }^{M\,I}\right)}^{2}}{\Delta^{2}}\right) & \left|\Phi_{\xi }^{M\,I}\right|\leq \Delta \\ 0 & \left|\Phi_{\xi }^{M\,I}\right|>\Delta \end{array}\right.$$

The elasticity tensor of that shell element is comprised of *D*_*b*_ and *D*_*s*_ shown in (14), and the target Poisson’s ratio is mainly related to *D*_*b*_. Therefore, the effective elasticity tensor of the microstructure *DH ijkl*(*DbH ijkl*, *D*_*s*_) is assembled by the effective elasticity tensor *DbH ijkl* and the constant *D*_*s*_. After that, the global stiffness matrix *K* in macro design domain can be calculated by *DH ijkl*. Here, the optimized microstructure is defined as isotropic or orthotropic material. Thus, there are two Poisson’s ratios *μ_1_* and *μ_2_* defined in the micro objective function, and they can be obtained by *DbH 11*, *DbH 12*, *DbH 22*, which are specific values of *DbH ijkl*, shown in Eq. (23).

(23) $$\begin{array}{c} \mu_{1}=D_{12}^{b\,H}\,\left(\alpha_{\xi }^{M\,I}\right)\,/\,D_{11}^{b\,H}\,\left(\alpha_{\xi }^{M\,I}\right)\\ \mu_{2}=D_{12}^{b\,H}\,\left(\alpha_{\xi }^{M\,I}\right)\,/\,D_{22}^{b\,H}\,\left(\alpha_{\xi }^{M\,I}\right) \end{array}$$

In the above formulas, *ΦMI ξ* is the level set function in the micro design domain *ΩMI ξ*. It can be calculated using spatial variables in the curvilinear coordinates [*ξ*, η, ζ] and time variables *t* based on the CSRBF interpolation in Eq. (24)

(24) $$\Phi_{\xi }^{M\,I}\,\left(\xi ,t\right)=\varphi_{\xi }^{M\,I}\,{\left(\xi \right)}^{T}\cdot \alpha_{\xi ,n}^{M\,I}\,\left(t\right)$$

The effective elasticity tensor *DbH ijkl* can be obtained through the numerical homogenization method:

(25) $$D_{i\,j\,k\,l}^{b\,H}\,\left(\alpha_{\xi }^{M\,I}\right)=\frac{1}{\left|\Omega_{\xi }^{M\,I}\right|}\,\int_{\Omega_{\xi }^{M\,I}}\left(\varepsilon_{p\,q}^{0\,\left(i\,j\right)}-\varepsilon_{p\,q}^{*}\,\left(u_{\xi }^{M\,I\,\left(i\,j\right)}\right)\right)\,D_{p\,q\,r\,s}^{b}\,\left(\varepsilon_{r\,s}^{0\,\left(k\,l\right)}-\varepsilon_{r\,s}^{*}\,\left(u_{\xi }^{M\,I\,\left(k\,l\right)}\right)\right)\,H\,\left(\Phi_{\xi }^{M\,I}\,\left(\alpha_{\xi }^{M\,I}\right)\right)\,d\Omega_{\xi }^{M\,I}$$

where *Db pqrs* is the elasticity tensor of the solid material; *ε0 pq* is the test unit strain field, (1,0,0)*^*T*^*, (0,1,0)*^*T*^* and (0,0,1)*^*T*^* as for the 2D problem; *ε^∗^ pq* is the strain field related to the displacement *uMI ξ*, which can be calculated via finite element analysis using the periodic boundary conditions of the microstructure:

$$\int_{\Omega_{\xi }^{M\,I}}\left(\varepsilon_{p\,q}^{0\,\left(i\,j\right)}-\varepsilon_{p\,q}^{*}\,\left(u_{\xi }^{M\,I\,\left(i\,j\right)}\right)\right)\,D_{p\,q\,r\,s}^{b}\,\varepsilon_{r\,s}^{*}\,\left(w_{\xi }^{M\,I\,\left(k\,l\right)}\right)\,H\,\left(\Phi_{\xi }^{M\,I}\right)\,d\Omega_{\xi }^{M\,I}$$

(26) $$=0,\forall w_{\xi }^{M\,I\,\left(k\,l\right)}\in \bar{U}\,\left(\Omega_{\xi }^{M\,I}\right)$$

where *w* is the virtual displacement field. The bilinear energy and the linear load forms of the finite element analysis in the microscale can be descried as:

$$F_{\xi }^{M\,I}\,\left(u_{\xi }^{M\,I},w_{\xi }^{M\,I},\alpha_{\xi }^{M\,I}\right)=$$

(27) $$\int_{\Omega_{\xi }^{M\,I}}\varepsilon_{i\,j}^{*}\,\left(u_{\xi }^{M\,I}\right)\,D_{p\,q\,r\,s}^{b}\,\varepsilon_{k\,l}^{*}\,\left(w_{\xi }^{M\,I}\right)\,H\,\left(\Phi_{\xi }^{M\,I}\,\left(\alpha_{\xi }^{M\,I}\right)\right)\,d\Omega_{\xi }^{M\,I}$$

(28) $$L_{\xi }^{M\,I}\,\left(w_{\xi }^{M\,I},\alpha_{\xi }^{M\,I}\right)=\int_{\Omega_{\xi }^{M\,I}}\varepsilon_{i\,j}^{0\,\left(i\,j\right)}\,D_{p\,q\,r\,s}^{b}\,\varepsilon_{k\,l}^{*}\,\left(w_{\xi }^{M\,I}\right)\,H\,\left(\Phi_{\xi }^{M\,I}\,\left(\alpha_{\xi }^{M\,I}\right)\right)\,d\Omega_{\xi }^{M\,I}$$

The bilinear energy and the linear load forms in the macroscale can be described as:

(29) $$F_{\xi }^{M\,A}\,\left(u_{\xi }^{M\,A},w_{\xi }^{M\,A},D_{i\,j\,k\,l}^{H}\right)=\int_{\Omega_{\xi }^{M\,A}}\varepsilon_{i\,j}\,\left(u_{\xi }^{M\,A}\right)\,D_{i\,j\,k\,l}^{H}\,\varepsilon_{k\,l}\,\left(w_{\xi }^{M\,A}\right)\,d\Omega_{\xi }^{M\,A}$$

(30) $$L_{\xi }^{M\,A}\,\left(w_{\xi }^{M\,A}\right)=\int_{\Omega_{\xi }^{M\,A}}p\,w_{\xi }^{M\,A}\,d\Omega_{\xi }^{M\,A}+\int_{\Omega_{\xi }^{M\,A}}\tau \,w_{\xi }^{M\,A}\,d\Gamma_{\xi }^{M\,A}$$

where *p* is the body force and *τ* is the traction of the boundary *ΓMA* ξ in the macroscale.

### Design Sensitivity Analysis

Based on the above concurrent topology optimization model, the sensitivity of the objective function can be obtained. Because of two scales, the sensitivity is divided into two parts and calculated through the first-order derivatives of the objective functions with respect to the expansion coefficients *αMIξ.* The sensitivity in the macro scale is:

(31) $$\begin{array}{c} \frac{\partial J^{M\,A}}{\partial \alpha_{\xi }^{M\,I}}=\frac{1}{2}\,\int_{\Omega_{\xi }^{M\,A}}{\left(u_{\xi }^{M\,A}\right)}^{T}\,\frac{\partial K_{\xi }^{M\,A}\,\left(D_{i\,j\,k\,l}^{H}\,\left(D_{i\,j\,k\,l}^{b\,H}\,\left(\alpha_{\xi }^{M\,I}\right),D_{s}\right)\right)}{\partial \alpha_{\xi }^{M\,I}}\,u_{\xi }^{M\,A}\,d\Omega_{\xi }^{M\,A}\\ =\frac{1}{2}\,\int_{\Omega_{\xi }^{M\,A}}\varepsilon_{i\,j}^{T}\,\left(u_{\xi }^{M\,A}\right)\,\frac{\partial D_{i\,j\,k\,l}^{H}\,\left(D_{i\,j\,k\,l}^{b\,H}\,\left(\alpha_{\xi }^{M\,I}\right),D_{s}\right)}{\partial \alpha_{\xi }^{M\,I}}\,\varepsilon_{k\,l}\,\left(u_{\xi }^{M\,A}\right)\,d\Omega_{\xi }^{M\,A} \end{array}$$

The effective elasticity tensor *DH ijkl* is comprised of two parts, where *DbH ijkl* is the function of design variables and *D*_*s*_ is constant. Hence, the first-order derivatives of *DH ijkl* can be calculated by:

(32) $$\frac{\partial D_{i\,j\,k\,l}^{H}\,\left(D_{i\,j\,k\,l}^{b\,H}\,\left(\alpha_{\xi }^{M\,I}\right),D_{s}\right)}{\partial \alpha_{\xi }^{M\,I}}=\frac{\partial D_{i\,j\,k\,l}^{b\,H}}{\partial \alpha_{\xi }^{M\,I}}$$

Then this sensitivity is utilized to calculate the first-order derivatives of *DbH ijkl* with respect to the design variables. Based on the shape derivative, the first-order derivatives of *DbH ijkl* with respect to the pseudo time *t* is:

$$\frac{\partial D_{i\,j\,k\,l}^{b\,H}}{\partial t}=\frac{1}{\left|\Omega_{\xi }^{M\,I}\right|}\,\int_{\Omega_{\xi }^{M\,I}}\left(\varepsilon_{p\,q}^{0\,\left(i\,j\right)}-\varepsilon_{p\,q}^{*}\,\left(u_{\xi }^{M\,I\,\left(i\,j\right)}\right)\right)\,D_{p\,q\,r\,s}^{b}$$

(33) $$\left(\varepsilon_{r\,s}^{0\,\left(k\,l\right)}-\varepsilon_{r\,s}^{*}\,\left(u_{\xi }^{M\,I\,\left(k\,l\right)}\right)\right)\,v_{\xi }^{n}\,\left|{\left(\nabla \Phi_{\xi }^{M\,I}\right)}^{T}\right|\,\delta \,\left(\Phi_{\xi }^{M\,I}\right)\,d\,\Omega_{\xi }^{M\,I}$$

*v n ξ*in Eq. (19) can be substituted in Eq. (33):

$$\frac{\partial D_{i\,j\,k\,l}^{b\,H}}{\partial t}=(\frac{1}{\left|\Omega_{\xi }^{M\,I}\right|}\int_{\Omega_{\xi }^{M\,I}}\left(\varepsilon_{p\,q}^{0\,\left(i\,j\right)}-\varepsilon_{p\,q}^{*}\left(u^{M\,I\,\left(i\,j\right)}\right)\right)D_{p\,q\,r\,s}^{b}$$

(34) $$\left(\varepsilon_{r\,s}^{0\,\left(k\,l\right)}-\varepsilon_{r\,s}^{*}\left(u^{M\,I\,\left(k\,l\right)}\right)\right)\varphi_{\xi }^{M\,I}{\left(\xi \right)}^{T}\delta \left(\Phi_{\xi }^{M\,I}\right)d\Omega_{\xi }^{M\,I})\dot{\alpha }{}_{\xi ,n}{}^{M\,I}(t)$$

The first-order derivatives of *DbH ijkl* with respect to *t* can also be calculated using the chain rule:

(35) $$\frac{\partial D_{i\,j\,k\,l}^{b\,H}}{\partial t}=\frac{\partial D_{i\,j\,k\,l}^{b\,H}}{\partial \alpha_{\xi }^{M\,I}}\,{\dot{\alpha }}_{\xi ,n}^{M\,I}\,\left(t\right)$$

Comparing Eqs (34) and (35), the first-order derivatives of *DbH ijkl* with respect to the expansion coefficients *αMIξ* can be given by:

$$\frac{\partial D_{i\,j\,k\,l}^{b\,H}}{\partial \alpha_{\xi }^{M\,I}}=\frac{1}{\left|\Omega_{\xi }^{M\,I}\right|}\,\int_{\Omega_{\xi }^{M\,I}}\left(\varepsilon_{p\,q}^{0\,\left(i\,j\right)}-\varepsilon_{p\,q}^{*}\,\left(u_{\xi }^{M\,I\,\left(i\,j\right)}\right)\right)\,D_{p\,q\,r\,s}^{b}$$

(36) $$\left(\varepsilon_{r\,s}^{0\,\left(k\,l\right)}-\varepsilon_{r\,s}^{*}\,\left(u_{\xi }^{M\,I\,\left(k\,l\right)}\right)\right)\,\varphi_{\xi }^{M\,I}\,{\left(\xi \right)}^{T}\,\delta \,\left(\Phi_{\xi }^{M\,I}\right)\,d\,\Omega_{\xi }^{M\,I}$$

Based on Eq. (36), the sensitivity in the micro scale is also obtained by:

$$\frac{\partial J^{M\,I}}{\partial \alpha_{\xi }^{M\,I}}=\frac{\partial {\left(\mu_{1}+1\right)}^{2}}{\partial \alpha_{\xi }^{M\,I}}+\frac{\partial {\left(\mu_{2}+1\right)}^{2}}{\partial \alpha_{\xi }^{M\,I}}$$

(37) $$=\frac{\partial {\left(D_{12}^{b\,H}/D_{11}^{b\,H}+1\right)}^{2}}{\partial \alpha_{\xi }^{M\,I}}+\frac{\partial {\left(D_{12}^{b\,H}/D_{22}^{b\,H}+1\right)}^{2}}{\partial \alpha_{\xi }^{M\,I}}$$

And the derivatives of the volume constraint with respect to the design variables can be calculated by:

(38) $$\frac{\partial V}{\partial \alpha_{\xi }^{M\,I}}=\int_{\Omega_{\xi }^{M\,I}}\varphi_{\xi }^{M\,I}\,{\left(\xi \right)}^{T}\,\delta \,\left(\Phi_{\xi }^{M\,I}\,\left(\alpha_{\xi }^{M\,I}\right)\right)\,d\Omega_{\xi }^{M\,I}$$

### Numerical Procedure

**FIGURE 3:**
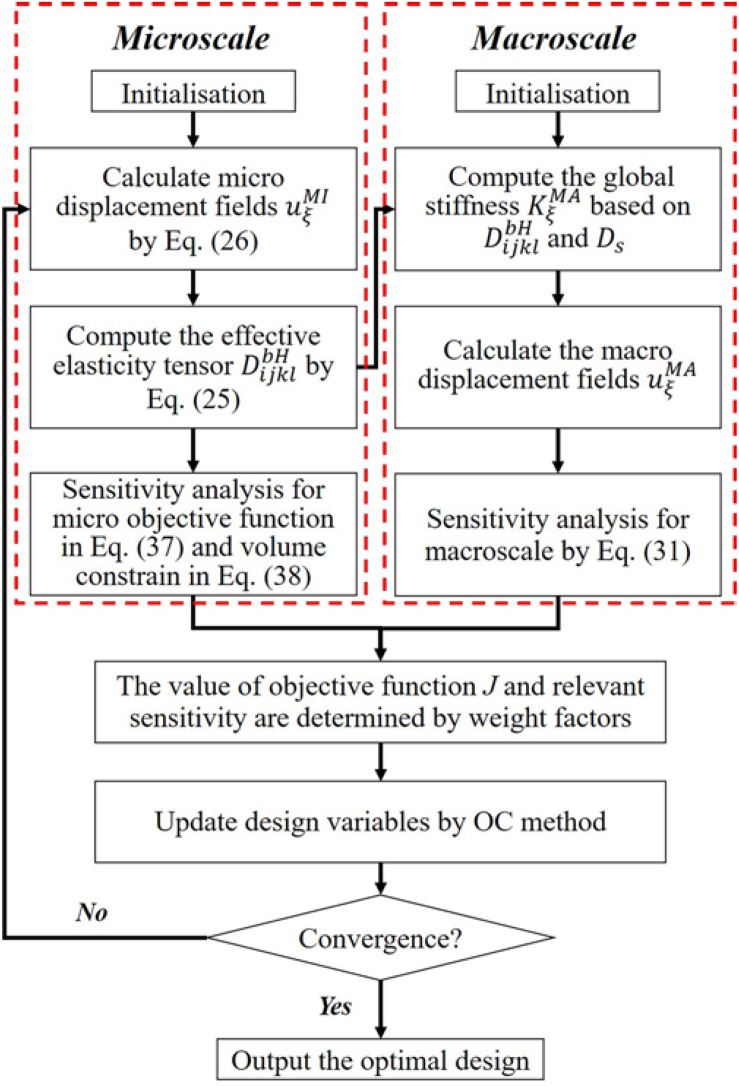
The flowchart of the concurrent topology optimization.

The flowchart of the proposed optimization method is given in Figure 3. At first, the micro displacement fields *uMI ξ* is obtained by solving the equilibrium equation Eq. (26). Then, the effective elasticity tensor *DbH ijkl* can be computed by using numerical homogenization method in Eq. (25). After that, the value of the micro objective function *J*^*MI*^ in Eq. (20) is calculated. The sensitivity of *J*^*MI*^ and the micro volume constraint with respect to design variables are obtained in Eqs (37) and (38), respectively. Simultaneously, the effective *DbH ijkl* and the constant *D*_*s*_ are utilized to compute the global stiffness matrix *K* of the macro structure, and then the macro equilibrium equation can be solved to get the macro displacement field *uMA ξ*. The macrostructural compliance and the derivative of *J*^*MA*^ are then calculated by *J*^*MA*^ in Eqs (20) and (31), respectively. Based on defined weight factors *W*_1_ and *W*_2_, the value of objective function *J* and relevant sensitivity can be determined. After that, the OC method is adopted to update design variables. The loop of the optimization is performed until the convergent criterion is satisfied.

## Numerical Results

**FIGURE 4:**
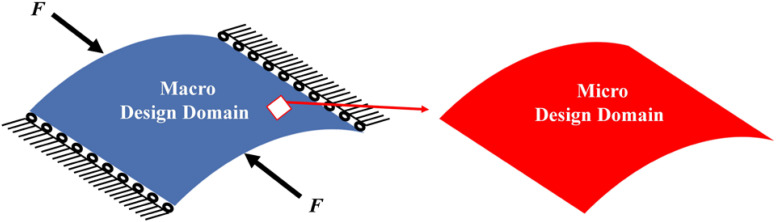
**(A)** Macro design domain; **(B)** micro design domain.

The concurrent topology optimization is implemented with MATLAB to obtain the micro-structured cellular composite structure with auxetic deformation. In the process, a piece of thin-walled structure is adopted as the macro design domain, which is indicated in blue color in Figure 4, subject to the loading and boundary conditions. The displacement of the stent along the circumference is fixed, while two unit forces are applied on the left and right edges in the axial direction. Meanwhile, the micro design domain is indicated in red color in Figure 4. Considering the computational efficiency and accuracy, the macro design domain is discretized by 30 × 30 shell elements with four nodes, where each element has a unit length, height. The micro design domain is discretized by 50 × 50 shell elements.

Coronary Stents are tube-shaped devices with close-cell units to keep the clogged arteries open. To adapt to a range of different arterial shapes, stents should have enough flexibility to accommodate turns or angles. Therefore, no matter what kind of coronary stents, most of them have low volume fractions of materials. Different volume fractions in the optimization can lead to different results with different negative Poisson’s ratios. In this design, 35% volume fraction is adopted at first. Then, the subsequent designs with smaller volume fractions will be determined based on the evaluation of the design with 35% material. To discuss design results, two parameters *Mu1* and *Mu2* are defined:

(39) $$M\,u\,1=D_{12}^{b\,H}\,/\,D_{11}^{b\,H},M\,u\,2=D_{12}^{b\,H}\,/\,D_{22}^{b\,H}$$

where *DbH 11*, *DbH 12*, *DbH 22* are specific values within the effective elasticity tensor.

### The Result of 35% Volume

**FIGURE 5:**
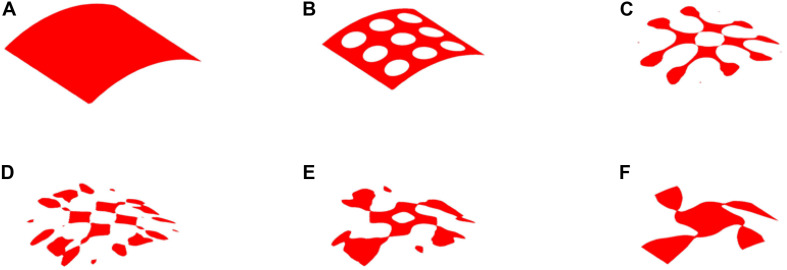
The optimization of 35% volume fraction: **(A)** Initial design; **(B–E)** four intermediate results; **(F)** final design.

**FIGURE 6:**
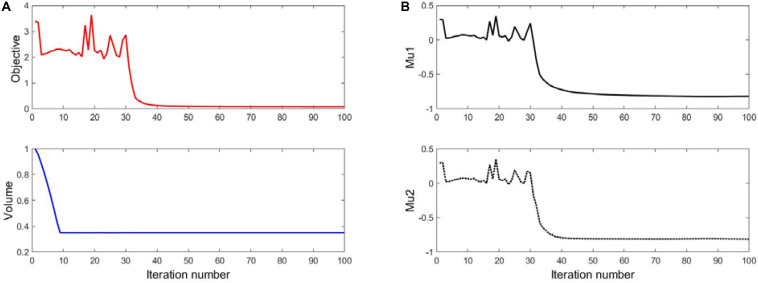
**(A)** The convergent histories of objective and volume; **(B)** Poisson’s ratios.

The optimization results with 35% volume fraction are presented in Figure 5. To track the dynamic change of the structural boundary during the optimization process, four intermediate results are used, as shown in Figures 5B–E), while Figures 5A,F are the initial design and final result, respectively. Except normal fluctuations from 10th to 30th optimization iterations, the objective function steadily minimized to close zero, and the convergence curve is shown in Figure 6A). The volume fraction of the structure is also steadily converged to 35%. These results indicate the proposed method is robust. The changes of the two Poisson’s ratios during the process are given in Figure 6B, where the results are *Mu1* = -0.8180, and *Mu2* = -0.8120. The two ratios are nearly “-1” close to the design objective, which shows that the method can effectively achieve a design with auxetics. However, the material distribution of the optimized structure is not uniform. The connections between the center and four branches are thinner than other parts, which can lead to non-uniform distribution of radial force. It will easily cause stent fracture at the thin connections, and result in a high incidence of ISR and ST. Besides that, the central region of the structure occupied by more than half materials without any gap, which may block side branches of arteries. Therefore, a smaller volume fraction 25% is then used to remove more materials from the thick branches and the center location.

### The Result of 25% Volume

**FIGURE 7:**
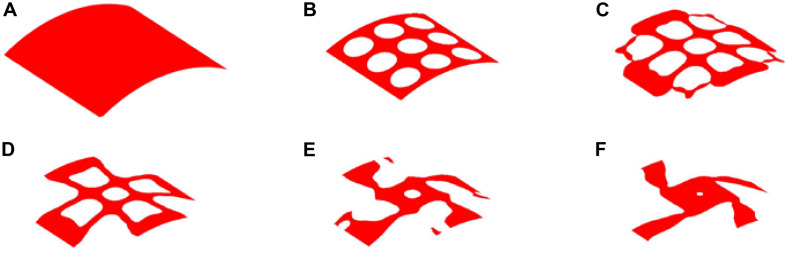
The optimization of 25% volume fraction: **(A)** Initial design; **(B–E)** four intermediate results; **(F)** final design.

**FIGURE 8:**
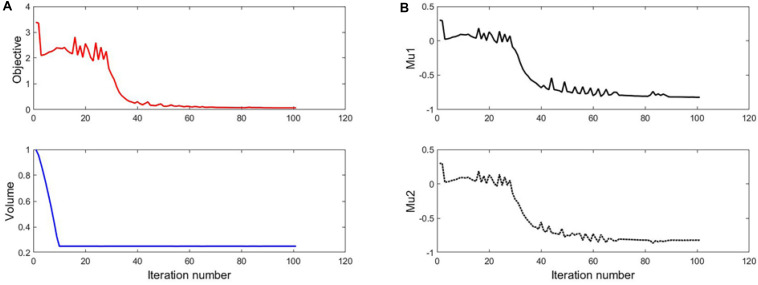
**(A)** The convergent histories of objective and volume; **(B)** Poisson’s ratios.

The optimization process of 25% volume fraction is then presented in Figure 7. And the convergence curves, similar to the design of 35%, can be found in Figure 8. The results of two Poisson’s ratios are *Mu1* = -0.8209, and *Mu2* = -0.8179, respectively. Compared with the result of 35% volume, in this case, the materials in the 4 branches and connections are better evenly distributed. However, the same issues still exist in the center of the structure, although a small hole is generated.

### The Result of 20% Volume

**FIGURE 9:**
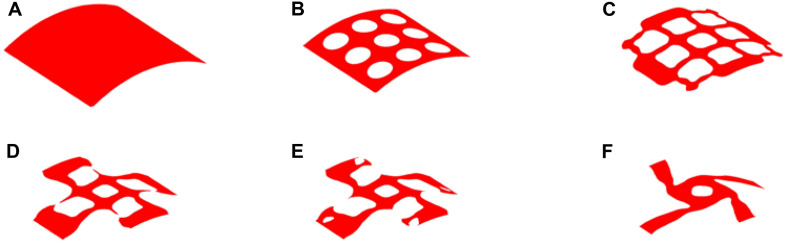
The optimization of 20% volume fraction: **(A)** Initial design; **(B–E)** four intermediate results; **(F)** final design.

**FIGURE 10:**
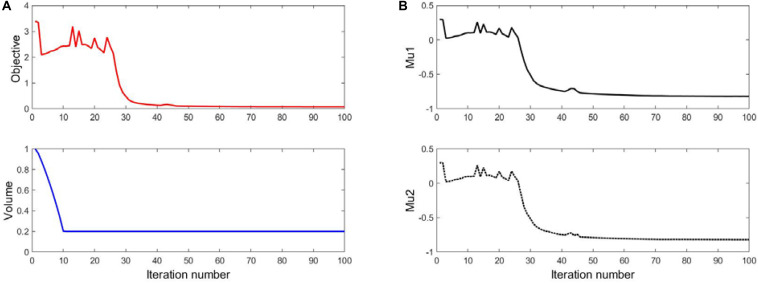
**(A)** The convergent histories of objective and volume; **(B)** Poisson’s ratios.

Therefore, 20% volume fraction is then used, aiming to removing more materials from the center position of the structure. The design results can be found in Figure 9, while the convergence curves are shown in Figure 10. The results of two effective Poisson’s ratios of the microstructure are *Mu1* = -0.8180, and *Mu2* = -0.8186.

**FIGURE 11:**
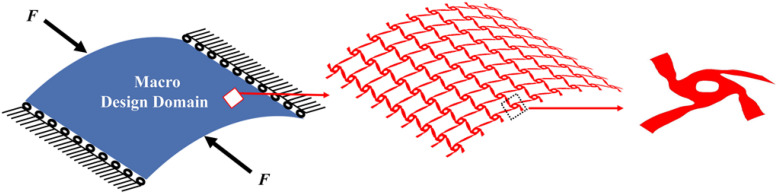
The final numerical design result.

Hence, based on the comparison of these three designs, it can be seen all of them have similar negative Poisson’s ratios at the peripheral and axial directions, and they are all close to the design objective. Nevertheless, the material distribution in the design of 20% is more uniform than others. It can provide a radial force that is better distributed to support vessels to prevent non-uniform expansion. The big hole in the center of the structure increase the gap of the stent to benefit blood flow from side branches of the arteries. Although less cover rate of the stent can reduce the biological rejection, a reasonable amount of materials can provide stronger and long-last support for the vessel and prevent higher incidence of complications caused by mechanical failures of the stent. Therefore, the third numerical design is adopted, as shown in Figure 11.

## Validation

From the numerical results, the topological design of auxetic stenting structures can overcome the limitations of conventional SE stents caused by the mechanical structural issues. Auxetic deformation has shown a great potential in enhancing the flexibility, conformability, and adaptability of the stent to prevent immediate injury, inadequate expansion, malapposition, and foreshortening issues, so as to reduce the incidence of ST and ISR. The topologically optimized design validations will be validated through numerical simulations with the commercial software ANSYS v2019R1.

### Simulation in ANSYS

**FIGURE 12:**
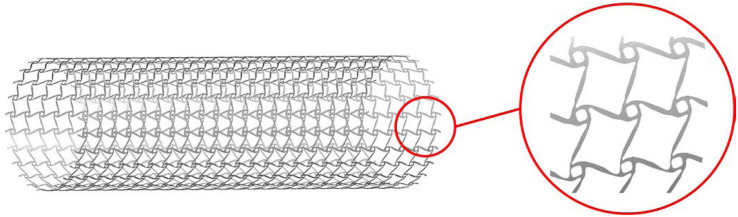
The geometry of the optimized stent.

To perform the simulation for the design, the geometry should be built based on the numerical design result. Firstly, the numerical design result (Figure 9) in MATLAB is output as a STL type file. Then, the STL file is imported into the software SpaceClaim which is integrated into ANSYS, to get the solid geometry for simulations. As mentioned in the design strategy, the optimized structure is the result with periodic microstructures, but it is hard in practice to use microstructures with very small scale by considering computational cost and manufacturing challenges. It is reasonable when considering the microstructures are actually independent of real dimensions but a relative scale. Therefore, the stenting architecture is assembled with 24 unit cells in the peripheral direction and 25 unit cells in the axial direction. Due to the use of the shell element, the geometry is modeled as a cylindrical surface with a specified thickness, which can help reduce the computational cost. The geometry of the optimized stent structure is illustrated in Figure 12. As the commonly used material in SE stents, Nitinol is utilized for the optimized stenting architecture in the simulation.

#### Simulation of NPR Behavior

**FIGURE 13:**
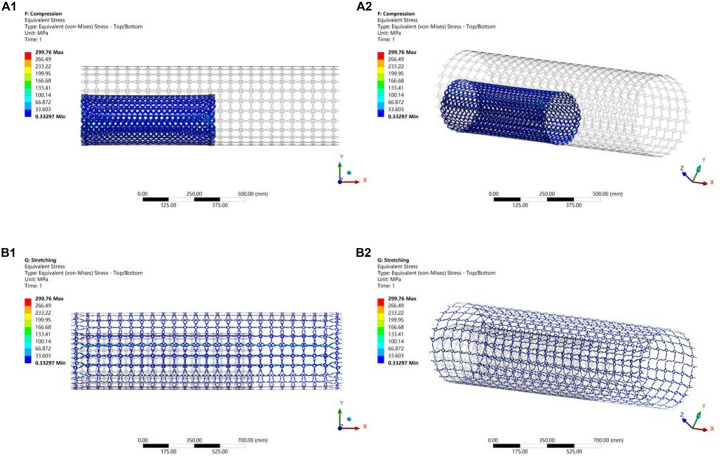
**(A)** Compression test; **(B)** Stretching test.

In that part, the compression and stretching tests are performed to validate the deformation mechanism of the optimized stenting architecture. The results are illustrated in Figure 13, where the gray outline wireframe shows the undeformed stent. With the compression, the NPR behavior can be easily found, which shows the size of the optimized stenting architecture becomes much smaller than undeformed shape in both peripheral and axial directions, which will benefit deliverability of the stent. Due to the stored strain energy from the elastic deformation, the stent structure can recover its undeformed shape via expansions in all directions when the compressed sent structure is released. Therefore, this deformation behavior can eliminate foreshortening when deploying the stent. The NPR behavior can also be demonstrated in the stretching test. The maximum equivalent stress in the validation model is 299.76 *Mpa*, much smaller than the yield stress of material Nitinol that is usually greater than 600 *Mpa*. Hence the new stenting structure has good strength to withstand circle loading in practice and low the risk of failure fracture.

#### Simulation of Inadequate Expansion and Malapposition

**FIGURE 14:**
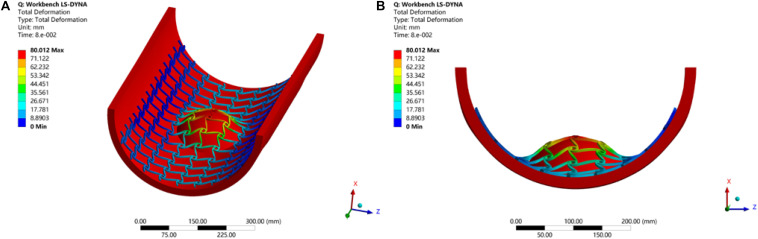
Stent expansion test: **(A)** 3D view; **(B)** front view.

Stents without desired flexibility and conformability may experience inadequate expansion and malapposition, to further result in an increased incidence of ST and ISR. Therefore, the simulation of the stent here is to test these mechanical performances of the optimized stenting architecture. The LSDYNA within ANSYS is utilized to simulate the expanding process for the stent. To reduce the computational cost, only a part of the stent is illustrated to simulate the expansion of the stent to the target vessel with a big plaque on the surface, as shown in Figure 14. From the result, it can be seen the stent adequately expands to cover the whole lesion, and adaptively deforms to fit the shape of the vessel with no gaps around the plaque. As we known, the stent malapposition can be described as gaps existing between the stent and the vessel wall. Hence, the current simulation result can demonstrate the optimized stent structure has excellent flexibility and conformability to prevent stent inadequate expansion and malapposition. Finally, all these benefits will help reduce the incidence of ST and ISR.

### 3D Printing

The additive manufacturing (3D printing) technology is particularly beneficial to the fabrication of solid geometries with complex shapes. Topologically optimized microstructures are often characterized with complex geometries, not compatible with most conventional manufacturing techniques. Hence it is a natural choice to implement topological designs with 3D printing methods. The prototype of the designed stenting architecture was enlarged by 15 times and then was printed using the Stratasys J750 machine at the ProtoSpace, the University of Technology Sydney. This machine can produce ultra-smooth surfaces and fine features with layer thickness as fine as 0.014 mm, to well represent the stent structure with auxetic microstructures.

**FIGURE 15:**
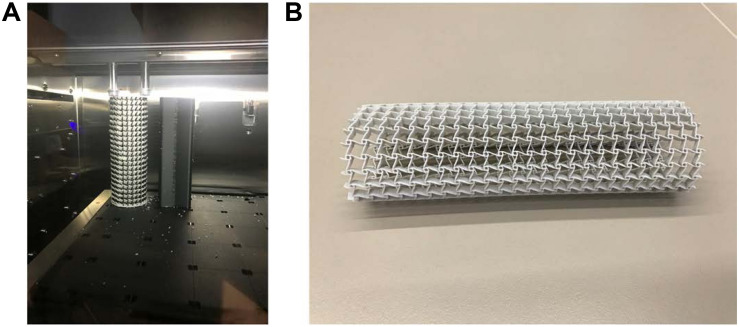
**(A)** printed in the printer Stratasys J750; **(B)** the prototype of the auxetic stent.

Topologically optimized designs often come with complex geometric shapes. How to manufacture the designs is also an important aspect that should be considered. In this work, due to the cost consideration and demonstration purpose, this paper employs a kind of plastic material for prototyping validation of the optimized design, rather than metallic materials, such as Nitinol. The material for the prototyping is composed of 30% Vero and 70% Tango, which can approximate the property of elastomer. The prototype is printed layer by layer following the axial direction as shown in Figure 15A. To avoid deformation of the structure during the printing, the prototype is supported by a solid cylinder filled inside with the same material. After that, the solid cylinder will be washed. The final prototype for the demonstration of the new stenting structure with 1.5 mm thickness is shown in Figure 15B. It is noted that any biocompatible materials can be used for production of the topologically optimized auxetic stents. In our near future work, the testing and characterization of the optimized auxetic SE stents with Nitinol will be conducted.

## Conclusion

This paper proposes a topology optimization method for the design of auxetic SE stents to reduce the risks of ST and ISR caused by mechanical or procedural factors of SE stents, such as inadequate stent expansion, stent fracture, and stent foreshortening and malapposition. The purpose of this paper is to reduce the incidence of ST and ISR of SE architectures, more from the mechanical structural and procedural aspects than the biological material aspect. However, the design factors of stents often influence the biological and even clinical outcomes. The importance of between stenting architectures and biological safety in the process of coronary artery disease has been shown in the study. The X-PLSM, in conjecture with numerical homogenization method, is used to establish a heuristic multiscale topology optimization approach for seeking novel auxetic stenting structural architecture. The main drawbacks of most current SE stents are expected to be avoided.

The numerical examples and simulations show that the topologically optimized structures offer auxetic deformation that can enable the stenting structures automatically and adaptively to deform (e.g., expansion). The unusual deformation mechanism will help overcome the inadequate stent expansion, stent fracture and stent malapposition particularly in self-expanding stents. The auxetic structures can also miniaturize the catheter enclosing the stent, which increases the deliverability of the stent system during the PCI procedure and avoids the immediate injure of the vessel. The stenting structures can also supply variable hoop strengths that will adapt to different radial forces when the artery cross-sectional shapes subject to change. When the shape getting smaller larger hooping strength, and vice versa, due to the enhanced indentation performance. The auxetic structures will also have a better capability to absorb vibration energy. In this work, topologically optimized architectures with auxetic metamaterials has been demonstrated to be able to overcome the drawbacks of self-expanding (SE) stents. Moreover, the proposed design optimization methodology and the auxetic cellular composite structures can also be extended to other biomedicine implants, such as esophageal stents, biliary stents, and femoropopliteal artery stents. It is noted that the blood flow also plays an important role in stent design performance. However, this manuscript mainly provides an opportunity to investigate how the unique mechanical properties gained from the optimized auxetic structure will benefit and help resolve inadequate stent expansion, stent fracture and stent malapposition in self-expanding stents. Hence, topologically optimized stents under the fluid dynamic consideration is outside the scope of this work, yet future studies expanding on the gained knowledge may reveal interesting further insights.

## Data Availability Statement

All datasets generated for this study are included in the article/supplementary material.

## Author Contributions

HX was a year-3 Ph.D. student who undertook the research and completed most detailed contents of this manuscript. ZL was the principal supervisor who has supplied the major research ideas, financial support, and writing of this manuscript and helped HX with the major tasks to complete this manuscript. TB was co-supervisor for HX and frequently attended the group meetings and provided useful advices and assistance particularly for 3D printing. SB has given a great help during the revision stage of this manuscript, including discussions of the reviewers’ comments, and the edit of the manuscript. All authors have made solid and important contributions to the completion of this manuscript.

## Conflict of Interest

The authors declare that the research was conducted in the absence of any commercial or financial relationships that could be construed as a potential conflict of interest.
